# Supramolecular super-helix formation *via* self-assembly of naphthalene diimide functionalised with bile acid derivatives

**DOI:** 10.1038/s41598-019-49235-5

**Published:** 2019-09-06

**Authors:** Sopan M. Wagalgave, Sachin D. Padghan, Mahesh D. Burud, Mohammad Al Kobaisi, Duong Duc La, Rajesh S. Bhosale, Sidhanath V. Bhosale, Sheshanath V. Bhosale

**Affiliations:** 1 Polymers and Functional Materials Division CSIR-Indian Institute of Chemical Technology, Hyderabad, 500007 Telangana India; 2 Academy of Scientific and Innovative Research (AcSIR), Ghaziabad, 201002 India; 3 School of Chemical Sciences, Goa University, Taleigao Plateau, Goa 403206 India; 4 Department of Chemistry and Biotechnology, FSET, Swinburne University of Technology, Hawthorn, VIC 3122 Australia; 5 Institute of Chemistry and Materials, 17 Hoang Sam, Cay Giay, Hanoi Vietnam; 6 Present Address: Department of Chemistry, Indrashil University, Kadi, Mehsana, 382740 Gujarat India

**Keywords:** Self-assembly, Molecular self-assembly

## Abstract

The design of chiral chromophores that lead to self-assembly of higher order helical structures is a powerful tool to understand the hierarchical helical structures of molecules of nature. In this work, we present a self-assembled helical super-structure produced *via* facial stacking of a bile acid bolaamphiphile derivative with a naphthalene diimide core (NDI-DCA), driven by solvophobic effects in THF–H_2_O solvent mixtures. The chirality of the helical microstructure is directed by the multiple chiral centres in the precursor molecule. The chirality of the hierarchical assemblies was observed using circular dichroism (CD), Scanning electron microscopy (SEM) and transmission electron microscopy (TEM) measurements. We propose that the NDI-DCA super-structures are formed *via* similar interactions and mechanisms to those observed in biological molecules such as proteins and DNA.

## Introduction

Helical molecular nanostructures such as deoxyribonucleic acid (DNA), and proteins such as collagen, found in many biological systems are product of the evolutionary processes of life^1,2^. These biological polymers are created from lower-order precursors that are either chiral or achiral, which assemble to form helical superstructures that perform precise and specific functions^3–5^. Amino acids are chiral building blocks that make up the primary structure of proteins using covalent bonds, which in turn fold into secondary structures to give helical three-dimensional nanostructures *via* non-covalent interactions^6^. In some instances, the self-assembly of such macromolecules leads to larger helical microstructures, as we see in tertiary structures such as collagen and DNA. Synthetic organic chiral molecules may interact selectively with biological chiral species, making chiral receptor for molecular recognition a field of growing interest. Chemist have utilised molecular design to produce specific chemical and physical properties, and detected the presence of chiral species selectively via spectroscopic^7^ or electrochemical^8^ signals.

Foldamers are a well-known example of self-assembled structures, with reported chiral microstructures having been reported. Recently, Nuckolls and co-workers described a chiral coral like self-assembly produced from −A−B−A−B− alternating electron donors (D = bithiophene) and acceptors (A = perylene diimide i.e. PDI)^9^. In another report^10^, a chiral shape-persistent, PDI-based nanoribbon was described, where three fused PDI monomers with intervening naphthalene subunits produced π-helix of helicene superstructures. In another example, in early 2016, Eiji and co-workers described in detais the development of supramolecular helical assemblies from achiral and chiral small molecule PDI foldamers^11^.

The Sanders group reported the formation of helical supramolecular nanotubes from naphthalene diimide (NDI) bearing amino acids via strong hydrogen-bonding in non-polar solvents and the solid state^12^. These helical tubes were also used as a receptor for fullerene molecules^13^. Although helical structures produce from donor-acceptor systems are very fascinating, having only donor or acceptor moieties with hydrophobic/hydrophilic side chains which can produce chiral superstructures in aqueous medium are rare.

NDIs have recently attracted much attention due to their tendency to form n-type semiconducting materials^14^, especially due to their ability to self-assemble *via* π-π stacking and form intricate controllable microstructures^15^. Thus, NDIs have became ideal candidates for supramolecular superstructure formation^16^, such as microflowers^17^, hydrogen-bonded helical nanotubes^12^, synthetic pores^18^, photoswitchable assemblies^19^, supramolecular photosystems with NDI as a donor and C60 as an acceptor^13^, self-sorted donor and acceptor chromophores^20^ are a few other similar examples.

Bile acid amphiphilic molecules are naturally occurring, and they comprise of an arched skeleton bearing opposing faces of hydrophobic and hydrophilic groups within the molecule^21^. The bile acid molecular structure consists of more than 10 chiral carbons, with –OH functional groups capable of Hydrogen bonding^22^. These chiral centres play an important role in the formation of helical supramolecular assemblies. Reported crystal structures of bile salts such as cholic acid show a resemblance to supramolecular chiral assemblies of proteins^23,24^. However, supramolecular self-assemblies of bile acids, which exhibit primary to secondary to tertiary structures that resemble protein formation in natural systems are relatively less studied^23–30^.

**Figure 1:**
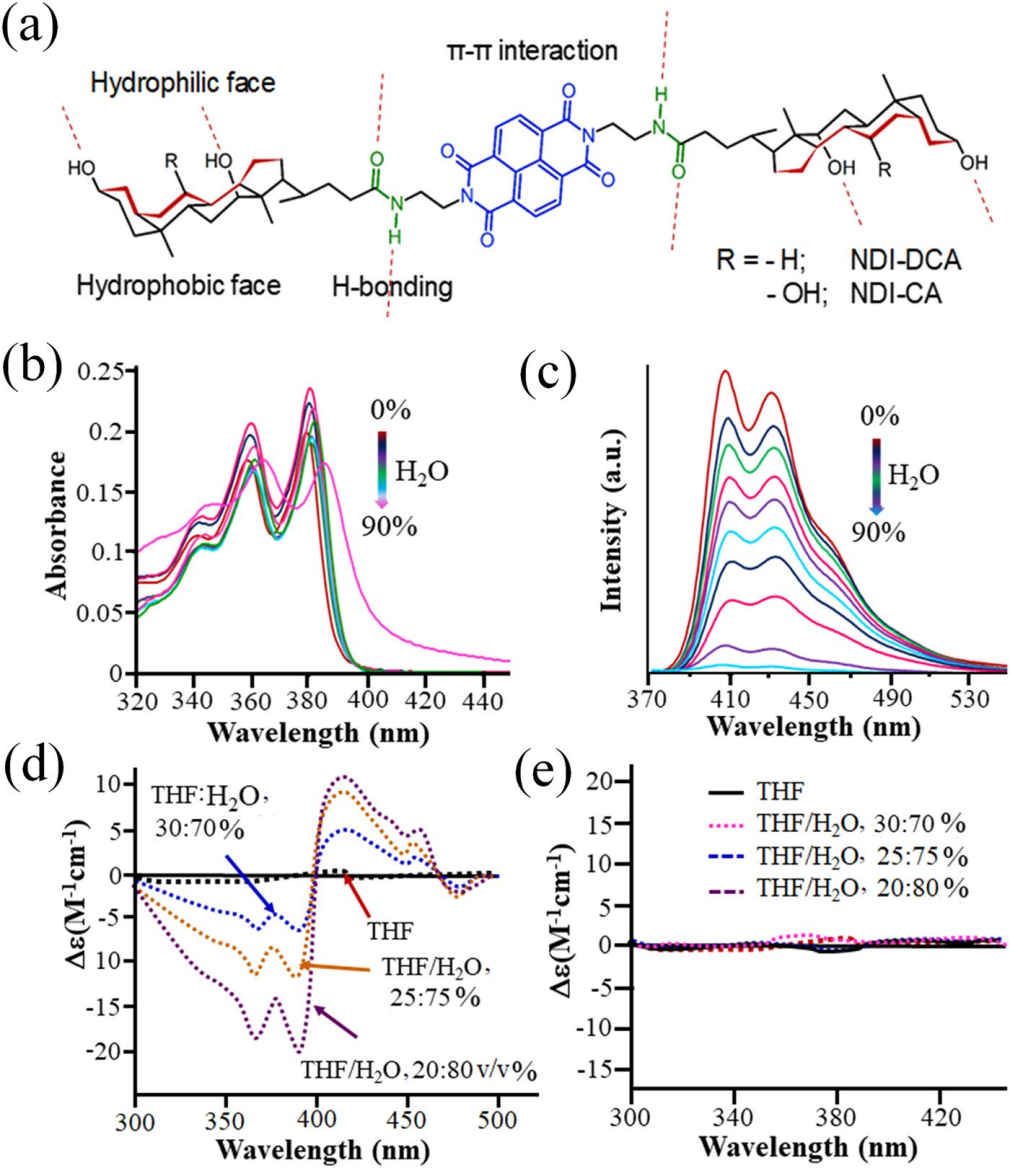
(**a**) Chemical structures of the synthesised NDIs bearing facial amphiphilic cholic acid head groups. (**b**) UV-vis spectra of NDI-DCA in THF (1 × 10^−5^ M) upon addition of water (0–90%). (**c**) Fluorescence spectra of NDI-DCA in THF (1 × 10^−5^ M) upon addition of water (0–90%) with λ_ex_ = 350 nm. CD spectra of (**d**) NDI-DCA and (**e**) NDI-CA at various ratios of water in THF.

Thus, we decided to conjugate NDI with bile acid and study the supramolecular self-assemblies by controlling solvophobic effects. Herein, we report the self-assembly of a cholic acid N-substituted NDI facial bolaamphiphile as shown in Fig. 1a. In a similar observation to that observed in steroid self-assembly^23,24^, here we found that the NDI-deoxycholic acid (NDI-DCA) facial bolaamphiphile self-assembled into supramolecular helical structures in THF–H_2_O solvent systems.

## Results and Discussion

### Synthesis

The naphthalenediimide-cholic acid compounds NDI-CA and NDI-DCA were synthesized from NDI-diamine and cholic or deoxycholic acids respectively, *via* amide coupling reactions in the presence of N-Ethyl-N′-(3-dimethylaminopropyl)carbodiimide hydrochloride (EDCI) and 1-hydroxybenzotriazole hydrate (HOBT) in dry DMF (for detail see experimental part). We confirmed the structure of both NDI-CA and NDI-DCA by means of FTIR, ^1^H and ^13^C NMR spectroscopy, as well as MALDI-TOF (ESI Figs S1–S9).

### UV-vis and fluorescence spectroscopy

NDI-CA and NDI-DCA are highly soluble in THF, but sparingly soluble in water. To prompt self-assembly of both NDI-CA and NDI-DCA, we have thus chosen water-THF mixtures. The UV-vis absorption spectra of NDI-DCA in THF displayed two intense peaks at 360 and 380 nm, which are typical of π-π* transitions of the NDI core, along with a shoulder peak at 340 nm (Fig. 1b). In order to investigate the effect of water on NDI-DCA self-assembly, the UV-vis spectra in THF–water solvent mixtures at various volumetric ratios was studied to see the effect of the water where the molecules are poorly soluble. The NDI-DCA absorption bands at 340, 360 and 380 nm all gradually decreased with an increasing water ratio in THF (0–80%) (Fig. 1b). However, at 90% water, the bands at 340, 360 and 380 nm red shifted by 10 nm to 350, 370 and 390 nm, respectively, indicating that NDI-DCA self-assembled with π-π stacking, affecting the π-π* electronic transitions of the NDI core. Similar absorption changes were observed for the NDI-CA compound, however the bathochromic shift of 10 nm in NDI-DCA is more pronounced than the 5 nm shift observed for all peaks of NDI-CA as shown in Fig. S10, which suggests a molecular interaction in the case of the NDI-DCA self-assembled structure. These bathochromic shifts in the UV-vis absorption bands suggest a *J*-type aggregation for both NDI-CA and NDI-DCA in THF – water solvent mixtures.

The fluorescence emission spectrum of NDI-DCA upon excitation at 350 nm in pure THF shows two emission peaks at 400 nm and 435 nm (Fig. 1c). The intensity of the emission bands of NDI-DCA decreased with the addition of water to THF solutions, with the PL intensity completely diminished at 90% water in THF. This implies that the addition of water to a THF solution of NDI-DCA induces self-assembled aggregation, *via* strong π-π stacking interactions of the NDI core causing quenching of the π conjugated core electronic relaxation transitions. Similarly, the PL study of NDI-CA in THF with the addition of water was carried out (Fig. S11), where upon excitation at 350 nm NDI-CA showed two emission peaks at wavelengths of 400 and 435 nm. The incremental addition of water up to 90% led to a gradual decrease in emission peak intensities, which completely quenched at 90% water in THF.

### Circular dichroism (CD)

Circular dichroism (CD) spectroscopy of NDI-DCA and NDI-CA in pure THF and three different water:THF ratios are shown in Fig. 1d,e, respectively. The CD signal of NDI-DCA is negligible in pure THF, but upon addition of water at 30% NDI-DCA exhibited a strong bisignate Cotton effect (CE) with two negative signals at 360 and 380 nm and a positive signal at 410 nm, with a *θ* = 0 crossing at 400 nm. The CD signals at 360 and 380 nm are consistent with the electronic absorption peaks (Fig. 1b). At 75 and 80% water in THF, the CD signal intensities increased significantly, suggesting chiral helical super-structure formation in NDI-DCA solutions (Fig. 1d). However, the CD signals of NDI-CA in THF and water in THF at 70, 75 and 80% solvent mixtures shows no CD active, even though the cholic acid is bearing 11 chiral centres (Fig. 1e). These low CD signals suggests that the molecular chirality of NDI-CA has not be transferred to form a helical assembly to amplify the CD response beyond the background chiral carbons in the precursor molecule. Similar results were observed in twisted ribbons generated by self-assembly of oligo(p-phenylene ethylene) bearing bile acid groups^29^.

### Computational methods

**Figure 2:**
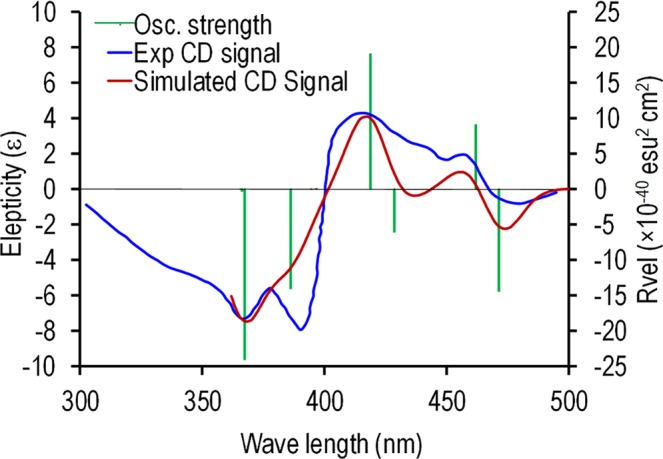
A comparison between the simulated and experimental CD spectra of NDI-DCA as calculated using TDDFT at B3LYP/def2-TZVP basis set.

*In vacuo* time dependent density functional theory (TDDFT) calculations were conducted using the ORCA 4.0 suit of programs^31^. Initially, the molecular structures were geometry optimized, then TDDFT calculations were carried out using the B3LYP def2-TZVP basis set. The Gauss-Sum 3.0 program^32^ and Avogadro version 1.2.0^33^ were used to visualise and process the results of ORCA calculations. The calculations revealed a LUMO concentrated on the NDI core for both NDI-CA and NDI-DCA, while the HOMO of NDI-CA is concentrated on the cholanamide moieties farthest away from the NDI core, and the HOMO of NDI-DCA is concentrated on amide links, closer to the NDI core, which may be due to having less electronegative oxygen atoms on the cholanamide moieties (see Fig. S12). Typical electronic transitions and similar *in vacuo* simulated density of state (DOS) UV and CD spectra were obtained for both molecules as shown in Fig. S13(A). The *in vacuo* simulated CD spectrum of NDI-DCA shows a 17 nm shift in comparison to the experimental results in solution, with the first 10 calculated excited states matching the experimental peaks. This red shift can be assigned to solvent mixture effects on the electronic structure of NDI-DCA. The simulated and experimental CD spectra were aligned using the crossing wavelength at 400 nm (See Fig. 2).

### Electron microscopy

**Figure 3:**
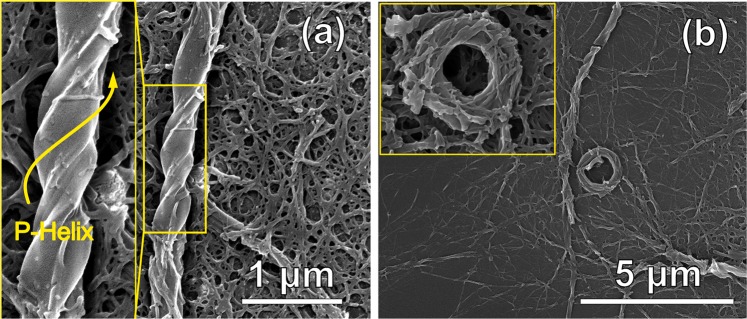
SEM images of NDI-DCA: (**a**) helical structures from 70% water in THF solution and (**b**) self-assembled rings from 75% water in THF solution.

Scanning electron microscopy (SEM) images allowed the visualization of the aggregates obtained from NDI-CA (Fig. S14) and NDI-DCA by solvent evaporation (Figs 3, S15–17) showing distinctive nanostructures at higher water – THF ratios. Spheres that were ~200–400 nm in diameter were observed for NDI-CA self-assemblies deposited from 80% water in THF solution (Fig. S14). These spheres are accompanied by a largely amorphous solid film. The spherical and film structures may be attributed to the bolaamphiphilic nature of NDI-CA, where highly hydrophilic cholic acid heads associated with water, and the hydrophobic cores associated together in an aqueous environment. The morphology of the self-assembled bolaamphiphile NDI-DCA was deposited from 70% water in THF solution, showing right-handed (*P*-type) helices alongside spherical microstructures 70–80 nm in diameter (Fig. S15). As shown in Fig. S15, the helical superstructures are several microns in length, and ~150–200 nm in width a helical pitch around 400–500 nm. At a higher water ratio of 75% solution, the helical structures tended to bundle to form complex rings and fibres (Figs 2, S16 and S17), to give bundles several microns in length and up to 500 nm in width, while the diameter of the ring superstructures is around 1.5 µm, and the inner hole is ~500 nm.

Importantly, **NDI-DCA** produced neither helical or coiled structures in THF:MeOH (20:80%) and THF:hexane (20:80%) nor in ACN:H_2_O (20:80%). Thus, it is crucial to use water as polar solvent to construct the supramolecular helical superstructures (See ESI Fig. S18).

**Figure 4:**
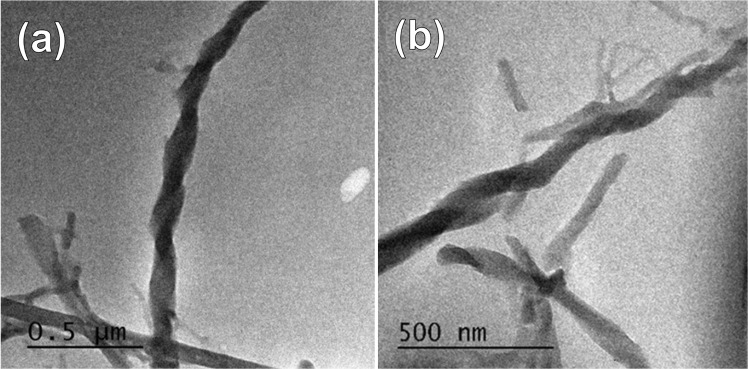
TEM images of aggregates of (**a**) right-handed helical structure of NDI-DCA in 70% water in THF and (**b**) zoomed image.

The helical morphology of NDI-DCA deposited from 70% water in THF was also confirmed using Transmission Electron Microscopy (TEM) showing a width of 100–200 nm and a pitch of ~400 nm (Fig. 4(a,b)).

### Dynamic light scattering

Dynamic light scattering (DLS) revealed that NDI-CA in 80% water in THF gives self-assemblies with an average hydrodynamic radius of around 531 nm in solution (Fig. S19a). This is in agreement with the spherical particles observed using SEM microscopy (Fig. S14). The self-assembly of NDI-DCA in 70 to 80% water in THF gave aggregates with a hydrodynamic radii (R_h_) of 341 nm at 75% water, decreasing to 295 nm at 80% water with a narrower distribution (Fig. S19a,b), suggesting small aggregates and dissolved species at higher fractions of water in THF^34^. These results are in agreement with perylene diimide (PDI) derivatives bearing various side-chains and the influence of aqueous solution on self-assembly. In theese studies it was shown that PDI bearing more hydrophilic moieties produce larger aggregates at low THF fractions in water, however the smaller hydrophilic chain on PDI required more THF in water^34^. Typically, DLS studies of PDI bearing hydrophilic oligoethylene chains on both the imide position show strong scattering compared to PDI bearing one imide, hydrophilic chains, and further imide hydrophobic chains. On the other hand, NDI-CA only shows a Rh ~340 nm in 80% water in THF. The formation of smaller aggregates at higher water content suggests that the features we observed using SEM and TEM are super-structures are grown from these smaller subunits observed using DLS upon solvent evaporation.

### Fourier-transform infrared spectroscopy (FTIR)

Conventional FTIR spectroscopy provided information about the self-assembled helical structures of NDI-DCA through observation of inter or intra molecular hydrogen bonding (Fig. S20). The NDI-DCA compound containing two amide bonds forms hydrogen bonding in 70, 75 and 80% water in THF. NDI-DCA showed strong, sharp vibrational bands at 1705 and 1665 cm^−1^, which were assigned to the symmetrical and asymmetrical stretching vibrational frequency of the carbonyl amide -NH-CO-R. However, in a 70% water in THF solution of NDI-DCA, the peak intensity decreased, along with the appearance of a band at 1641 cm^−1^. Upon further increasing the water percentage up to 80% water in THF, the peak intensity decreased along with a broadening of the peak and a shift of the carbonyl amide bond to 1639 cm^−1^. These results support our hypothesis, as the low-wavenumber shift of the amide bond observed in THF/water indicates a strong hydrogen-bonding interaction, with similar results obtained in solvent-polarity-tuned nanostructures of anthracene derivatives^35^.

### Time-resolved fluorescence lifetime measurements

Time-resolved fluorescence lifetime measurements were carried out on a picosecond time-correlated single photon counting (TCSPC) instrument. which allows determination of fluorescence lifetimes of molecules in the range of picoseconds to hundreds of nanoseconds^7^. Herein, we measured the fluorescence lifetime of NDI-DCA in various water:THF ratios. TCSPC studies of NDI-DCA in THF and 70, 75 and 80% water in THF solvent mixtures, using 350 nm as excitation and 431, 434, 432, and 432 emission wavelengths is shown in Fig. S21. It can be seen that a fluorescence decay lifetime is 0.965 ns (100%) in THF ias one component is observed in Table S1. Interestingly, the fluorescence lifetime of NDI-DCA in 70 and 75% water in THF also showed only one component, 0.949 ns (100%) and 0.937 ns (100%),. However, increasing the water ratio to 80% gives three components, 0.712 ns (26.16%), 0.004 ns (71.36%) and 2.67 ns (2.48%) decays. This significant decrease in fluorescence lifetime is evidence of self-assembly and aggregation of NDI-DCA upon increasing the water ratio in THF–water solvent mixtures.

## Discussion

Molecular design of NDI based molecules can produce specific arrangements resulting in micro and nanostructures with various geometries. This is dependent on the nature and size of the substitution on the NDI core. It has been reported that the N-alkane, peptide, aromatic and many other substitutions on the NDI can result in chiral arrangements and produces helical tubes^12,13^, twisted ribbons or helices with preferred handedness^36,37^.

**Figure 5:**
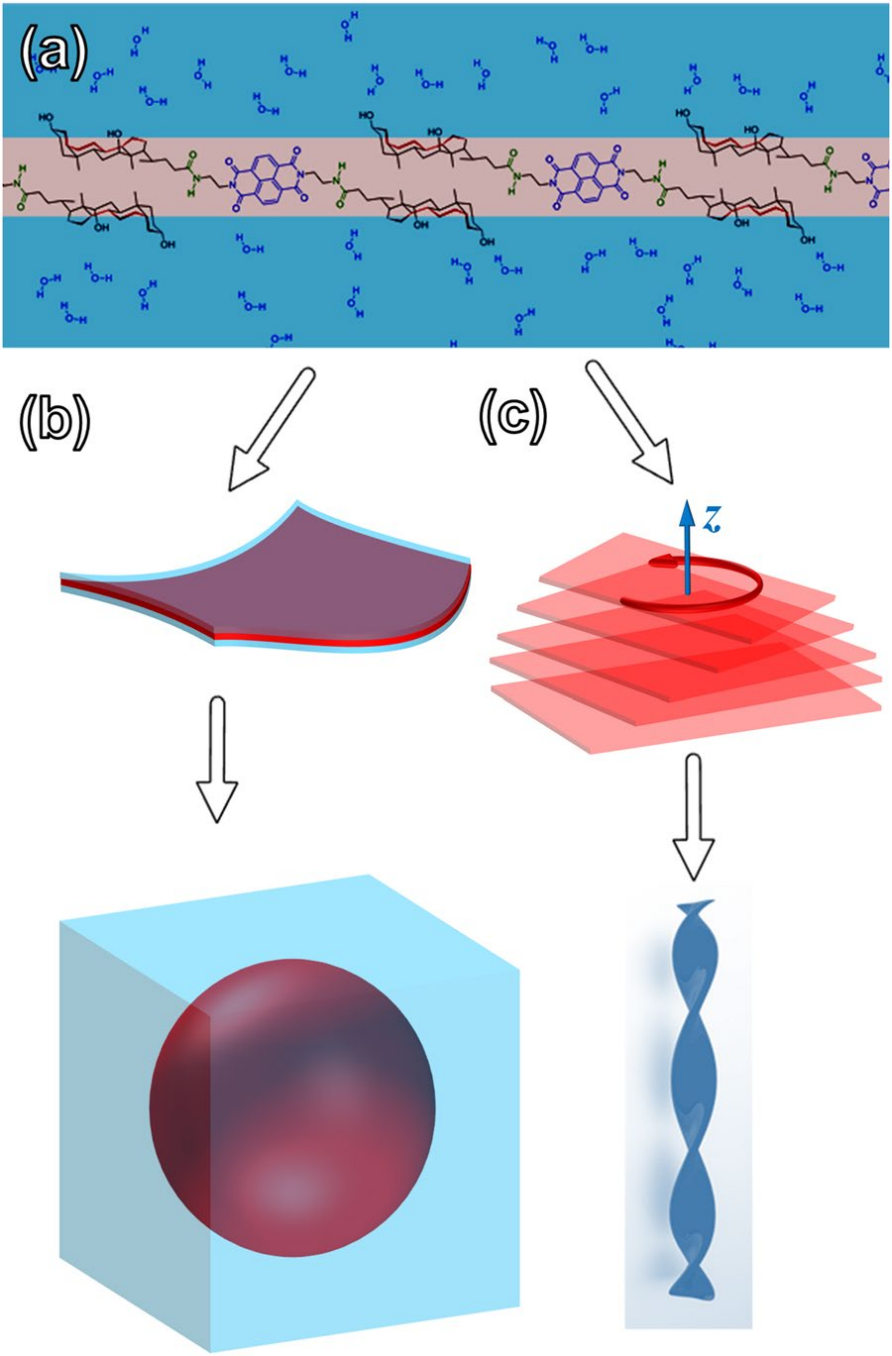
Schematic representation of (**a**) monolayer produced by NDI-DCA in highly aqueous environment, (**b**) micellar or spherical aggregate of NDI-DCA, and (**c**) the twisted stacked monolayer plates of NDI-DCA that gives right-handed chiral helix fibres.

We have established through the techniques above that the self-assembly of NDI-bile acid conjugates of NDI-CA and NDI-DCA are controlled by solvophobic effects. Our experiments have shown that the chirality in the molecular structures can be amplified to give chiral supramolecular microstructures. UV-vis and fluorescence emission spectra indicated that NDI-CA and NDI-DCA undergo *J*-type aggregation with increasing solvophobic effects. The CD spectra demonstrate that NDI-CA does not show Cotton effects with increasing the water ratio in THF – water solvent mixtures, which can be attributed to its extra hydroxyl group in the hydrophilic face of the cholic acid moieties, which reduces the solvophobic effect. An increasing water ratio and therefore leads to a lower degree of self-assembly in the z-axis (Fig. 5(c)). This is confirmed by the lack of twisted chiral micro-structures in SEM images of NDI-CA.

On the other hand, NDI-DCA surprisingly showed a progressively increasing CD signal with increasing water ratio in THF – water solvent systems. This amplification of the CD signal is induced by the progressive self-assembly of NDI-DCA due to solvophobic effects, where the molecular structure of the deoxycholic acid moieties have less hydrophilic functional groups. SEM images of NDI-DCA suggest that the chirality of the molecular structure is expressed in the handed helical microstructure *via* self-assembly. The SEM micrographs showed *P*-type helical micro-structures with spheres formed at 70% water in THF. Increasing the water ratio to 75% resulted in aggregation of the helical micro-structure sub-units to give super-helix and ring-like morphologies. This hierarchical aggregation trend increased further when the water ratio was increased to 80%. Based on these microscopic and spectroscopic observations, a mechanism is proposed to describe the NDI-DCA self-assembly process, which relies on (i) π-π stacking of the NDI core, (ii) van der Waals interactions on the hydrophobic face, (iii) H-bonding on the hydrophilic face of the deoxycholic acid moieties and (iv) H-bonding between the peptide linkages. As the water ratio in THF – water solutions of NDI-DCA increases, hydrophobic interactions between the NDI cores and the hydrophobic face of the deoxycholic acid moieties initially results in monolayer aggregates forming spherical micellar microstructures (See Fig. 5(a)).

The hydrogen bond double hook of both the NDI-CA and NDI-DCA molecular structures gives a twisted conformation, with handedness deduced from the chiral centers of the cholanamide moieties. Sada *et al*. showed that cholanamides can also accommodate other small solvent molecules in their self-assemblies and crystal structures. It can be theorized that the helix formation in the less hydrophilic NDI-DCA solution with increasing water ratio occurs via z-stacking of monolayers through the hydrophilic face due to increased hydrophobicity by loss of one of the hydroxyl groups in comparison to NDI-CA. This single structural difference changed the hydrophobic – hydrophilic balance to a degree that resulted in a fundamental shift in the self-assembly processes of the two molecules.

The formation of the microspheres and amorphous film from NDI-CA is evidence of irregular z-aggregation of the molecules driven by solvent evaporation, which is confirmed by NDI-CA weak CD spectra signals. This arrangement allows for the amide linkages and the hydroxyl groups to be solvated, while hydrophobic interactions of the CA moieties and the π-π stacking NDI core is fully utilised in the self-assembled sheet (Fig. 5(a,b)).

In summary, we have demonstrated control over self-assembly of bolaamphiphile molecules *via* molecular design. A small change in the NDI-cholic acid (NDI-CA) facial bolaamphiphile to give NDI-deoxycholic acid (NDI-DCA) has significantly changed the hydrophobic – hydrophilic balance of the molecule resulting in a fundamental shift in self-assembling behaviour using solvophobic effects. Increasing the hydrophobicity of the N-substitutions of the NDI core resulted in the formation of super-helical chiral architectures in high water ratios of water-THF solutions of NDI-DCA, while NDI-CA forms globular and amorphous aggregates. The chirality of the helical microstructure of NDI-DCA are an amplification of the chiral centres of the parent molecule as demonstrated by CD spectroscopy. Therefore, we have concluded that the chirality in the molecular structure at the atomic level has guided the self-assembly, to give the chirality in the microstructure, in a similar way as the proteins we observe in living organisms.

## Materials and Methods

### General methods & materials

**Figure 6:**
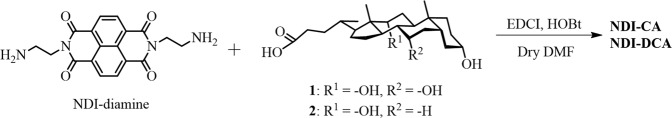
The synthetic pathway to NDIs bearing facial amphiphilic bile acid head groups.

Chemicals and reagents were purchased from Sigma-Aldrich, Bengaluru, Karnataka, India. All reagents were used as received without further purification. All air- and water-sensitive reactions were performed under nitrogen atmosphere. Thin layer chromatography (TLC) (Merck Co.) was performed using 0.25 mm thick plates pre-coated with silica gel (40–60 mm, F254) and visualized using UV light (254 and 365 nm). ^1^H NMR spectra were recorded with a 300 MHz, 400 MHz and 500 MHz on Bruker spectrometer using DMSO-d_6_ as solvent. Tetramethylsilane was used as an internal standard. FT-IR spectra were recorded on a Thermo Nicolet Nexus 670 FR-IR spectrometer in the form of non-hygroscopic KBr pellets. ESI-MS data were taken on Shimadzu lab solutions. High-resolution mass spectra (HRMS), atmospheric-pressure chemical ionization (APCI) experiments were carried out on FTMS, ionizing by APCI from an atmospheric solids analysis probe (ASAP). MALDI-TOF measurements were performed on a Schimadzu Biotech Axima spectroscopic instrument. The synthetic pathway to NDIs bearing facial amphiphilic bile acid head groups i.e. NDI-CA and NDI-DCA is shown in Fig. 6.

*Synthesis of (R,S,R,R,S,R,R,S,S,S,4R,4*′*R)-N,N*′*-((1,3,6,8-tetraoxobenzo [lmn][3,8]phenanthroline-2,7(1H,3H,6H,8H)-diyl)bis(ethane-2,1-diyl))bis(4-((3R,5S,7R,8R,9S,10S,12S,13R,14S,17R)-3,7,12-trihydroxy-10,13-dimethylhexadeca hydro-1H-cyclopenta[a]phenanthren-17-yl) pentanamide) (NDI-CA)*
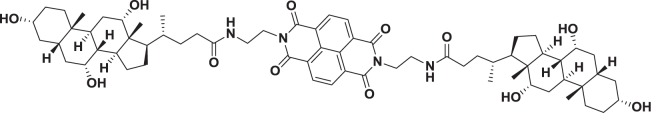


Cholic acid 255 mg (0.62 mmol), HOBt 167 mg (1.2 mmol) and 1-ethyl-3-(3-dimethylaminopropyl) carbodiimide 239 mg (1.2 mmol) were dissolved in dry DMF. The reaction mixture was stirred for 30 min at 0 °C. To this reaction mixture NDI-diamine 100 mg (0.28 mmol) was added. After complete addition of NDI-amine, the resulting reaction mixture was stirred at ambient temperature for further 24 h. The completion of reaction was monitored by TLC. The reaction mixture was poured in to crushed ice and stirred well yields white precipitate. The obtained white precipitate was filtered and purified by column chromatography on neutral Al_2_O_3_ (5% MeOH/CH_2_Cl_2_). FT-IR (KBr, cm^1^): υ 769, 1076, 1190, 1246, 1341, 1400, 1578, 1664, 1705, 2932, 3182, 3400; ^1^H NMR (DMSO-*d*_6_, 300 MHz): δ 8.68 (s, 4H; Ar-H) 7.89 (s, 2H; NH) 4.16 (t, *J* = 5.50 Hz, 4H) 3.67 (s, 3H) 3,59 (s, 3H) 3.18 (s, 4H) 2.22 to 1.19 (m, 54H) 0.79 (s, 12H: CH_3_) 0.44 (s, 6H; CH_3_); ESI-MS (*m/z %*): 1134.2 (100) [M+H]^+^; MALDI-TOF: 1156.17 [M+Na]^+^.

*Synthesis of (R,R,R,S,R,R,S,S,S,4R,4*′*R)-N,N*′*-((1,3,6,8-tetraoxobenzo[lmn] [3,8]phenanthr oline-2,7(1H,3H,6H,8H)-diyl)bis(ethane-2,1-diyl))bis(4-((3R,5R,8R,9S,10S,12S,13R,14S,17R)-3,12-dihydroxy-10,13-dimethylhexadecahydro-1H-cyclopenta[a]phenanthren-17-yl)pentana mide) (NDI-DCA)*
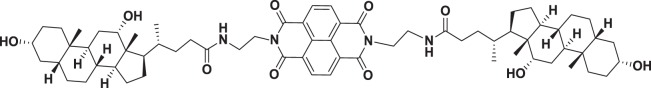


Deoxycholic acid 245 mg (0.62 mmol), HOBt 167 mg (1.2 mmol) and 1-ethyl-3-(3-dimethylaminopropyl)carbodiimide 239 mg (1.2 mmol) were dissolved in dry DMF. The reaction mixture was stirred at 0 °C for 30 min then NDI-diamine 100 mg (0.28 mmol) was added. The resulting reaction mixture was stirred at ambient temperature for 24 h. The completion of reaction was monitored using TLC. The reaction mixture was poured in to crushed ice, white precipitate was formed. The precipitate was filtered and the obtained crude product was purified by column chromatography on neutral Al_2_O_3_ (4% MeOH/CHCl_2_). FT-IR (KBr, cm^1^): 770, 1043, 1246, 1341, 1384, 1455, 1665, 1705, 2863, 2933 and 3411; ^1^H NMR (DMSO-*d*_6_, 300 MHz) δ: 8.68 (s, 4H; Ar-H) 7.90 (s, 2H; NH) 4.48 (d, *J* = 4.12 Hz, 2H) 4.16 (d, *J* = 4.12 Hz, 6H) 3.68 (s, 2H) 1.94 to 0.91 (m, 56H) 0.82 (s, 12H; CH_3_) 0.46 (s, 6H; CH_3_); ESI-MS (*m/z %*): −1102 (100) [M+H]^+^; HRMS: calculated for C_66_H_93_O_10_N_4_ = 1101.6886 Found = 1101.6889 [M+H]^+^; MALDI-TOF: 1123.66 [M+Na]^+^.

### Spectroscopic measurements

#### UV–Vis spectroscopy

UV–vis absorption spectra were recorded on a Shimidazu UV-1800 spectrophotometer at room temperature, spectrometer using 1 cm path length cuvette. A 0.2 mL aliquot of the stock solution of **NDI-DCA** or **NDI-CA** (conc. = 10^−3^ M) was transferred to THF, and made up to 2 mL volume. Similarly, 0.2 mL of **NDI-DCA** or **NDI-CA** was transferred to THF/water (with various ratios). Each time mixture was allowed to equilibrate for 2 h prior to the spectroscopic measurements.

#### Fluorescence spectroscopy

Fluorescence emission spectra were recorded on an RF-6000 (Schimadzu, Japan) spectrofluorophotometer. All experiments were performed in a quartz cell with a 1 cm path length with 360 nm excitation wavelength. (λ_ex_ = 350 nm).

#### Circular Dichroism

CD spectra were recorded on an AVIV 202 CD spectrometer under a nitrogen atmosphere. The measurements were performed in a quartz cuvette with a 1 mm path length over the range of 330–430 nm in THF:H_2_O solution, similar to UV-vis absorption.

#### Scanning electron microscopy (SEM) imaging

SEM samples were prepared by solvent evaporation on a silicon wafer and then sputter coated with gold for 10 s at 0.016 mA Ar plasma (SPI, West Chester, USA) for SEM imaging using a FEI Nova NanoSEM (Hillsboro, USA) operating at a high vacuum which provided direct visualisation of the self-assembled aggregated structures.

#### Transmission electron microscopy (TEM)

The samples were prepared by evaporating sample solvents on a holey carbon grid and the images were taken on a JEOL 1010 100 kV TEM.

#### DLS measurements

were conducted using a Brookhaven Instrument Corp., 90 Plus Particle Size equipped with a He–Ne laser (632.8 nm, 35 mW) and quartz cuvette. The SEM imaging was performed using a FEI Nova NanoSEM (Hillsboro, USA) operating at high vacuum at the voltage of 15 keV.

#### Molecular modeling

Density functional theory (DFT) calculations with no consideration of dispersion interactions in gas phase was conducted using Gaussian 09 suite of programs.

#### Fluorescence lifetime measurements

were carried on a picosecond time-correlated single photon counting (TCSPC) setup (FluoroLog3-Triple Illuminator, IBH Horiba JobinYvon) employing a picosecond light emitting diode laser (NanoLED, λex = 350 nm). The samples for the analyses were prepared in THF and THF:Water evaluated using a 1 cm cuvette at 25 °C.

## Supplementary information

Supplementary Material
