# Supplementary material for: Supramolecular super-helix formation via self-assembly of naphthalene diimide functionalised with bile acid derivatives
Source: Sci Rep. 2019 Sep 6;9:12825. doi: 10.1038/s41598-019-49235-5 (PMC6731272; doi:10.1038/s41598-019-49235-5)
Supplement: Supplementary file 1 — Supplementary Material [file 41598_2019_49235_MOESM1_ESM.pdf]

Supplementary Information

Supramolecular super-helix formation *via* self-assembly of naphthalene diimide functionalised with bile acid derivatives

Sopan M. Wagalgave,[1,2] Sachin D. Padghan,[1] Mahesh D. Burud,[3] Mohammad Al Kobaisi,[4] Duong Duc La,[5] Rajesh S. Bhosale,[1,†] Sidhanath V. Bhosale,[1,2*] Sheshanath V. Bhosale[3*]

[1]Polymers and Functional Materials Division CSIR-Indian Institute of Chemical Technology, Hyderabad 500007, Telangana, India. [2]Academy of Scientific and Innovative Research (AcSIR), Ghaziabad-201002, India. [3]School of Chemical Sciences, Goa University, Taleigao Plateau, Goa-403206, India. [4]Department of Chemistry and Biotechnology, FSET, Swinburne University of Technology, Hawthorn VIC 3122, Australia. [5]Institute of Chemistry and Materials, 17 Hoang Sam, Cay Giay, Hanoi, Vietnam. [†]Present Address: Department of Chemistry, Indrashil University, Kadi, Mehsana-382740, Gujarat, India. *Correspondence and requests for materials should be addressed to S.V.B. (email: svbhosale@unigoa.ac.in) or S.V.B. (CSIR-IICT): bhosale@iict.res.in

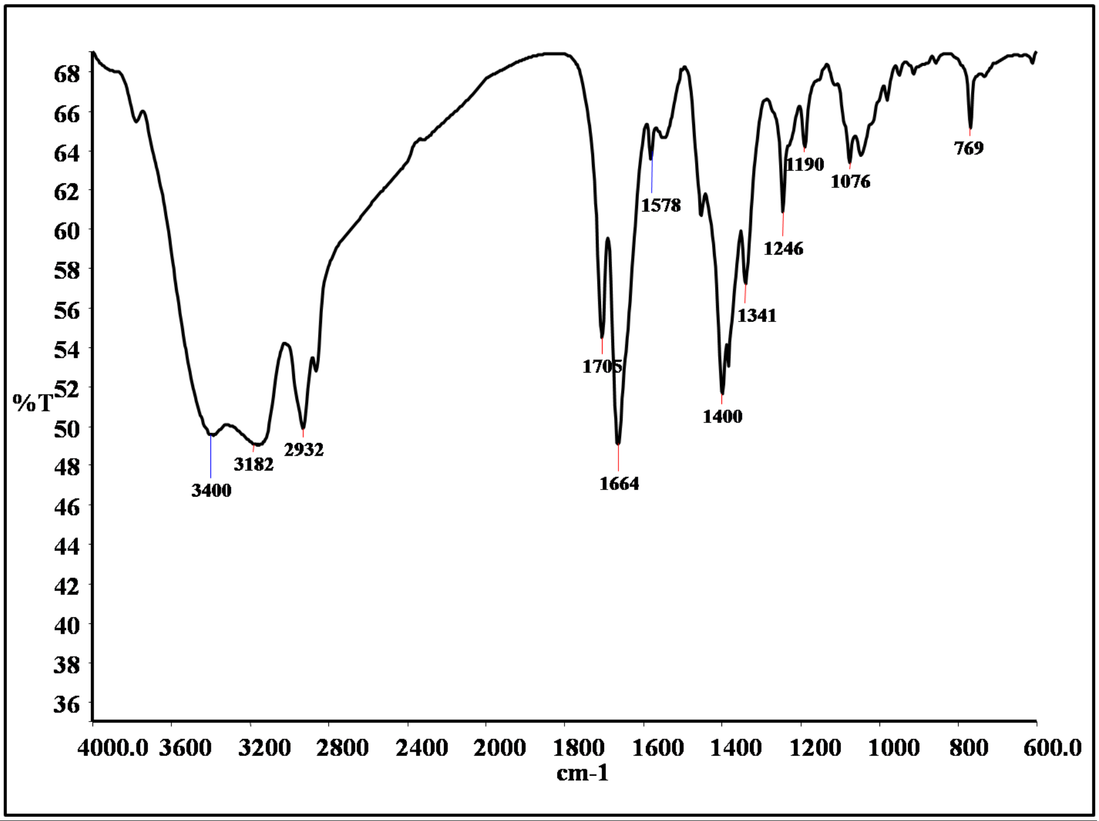

Figure S1. FT-IR spectra of **NDI-CA**.

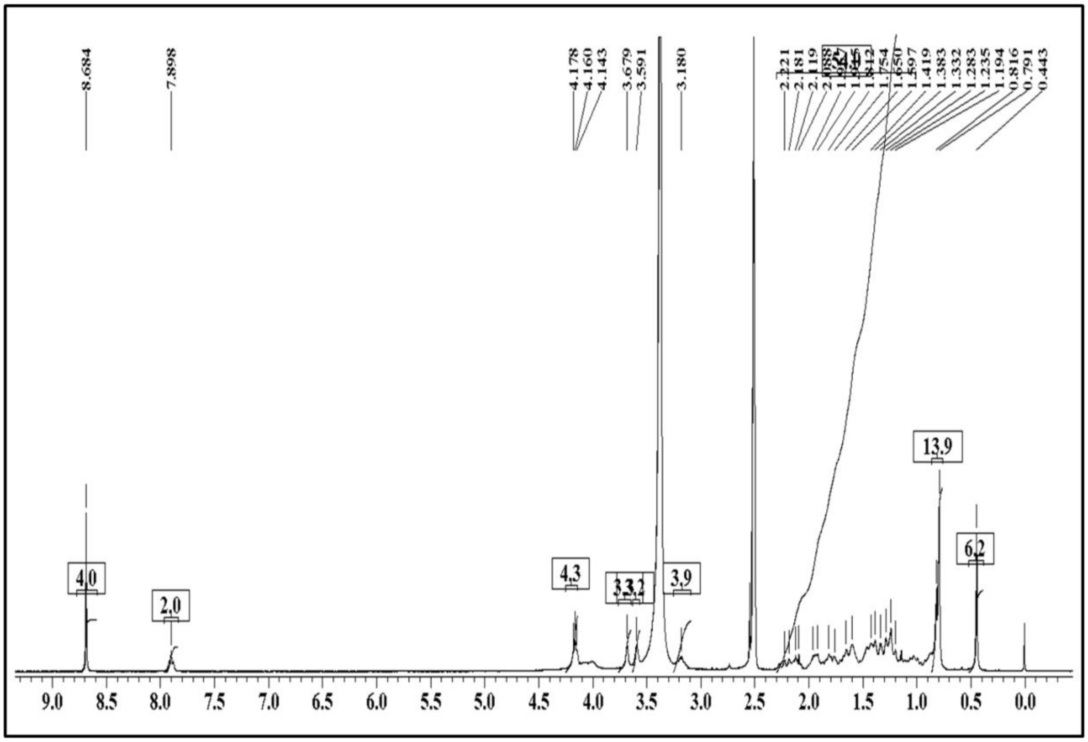

Figure S2. ^{1}H NMR spectra of **NDI-CA**.

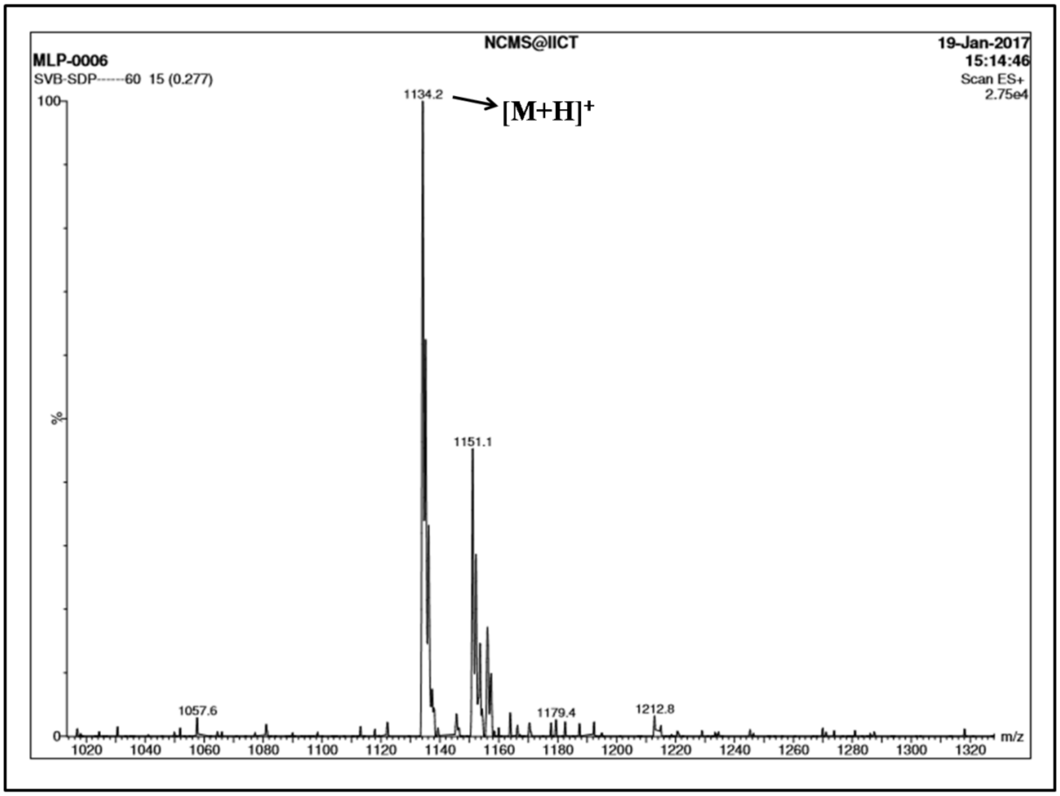

Figure S3. ESI mass spectra of **NDI-CA**.

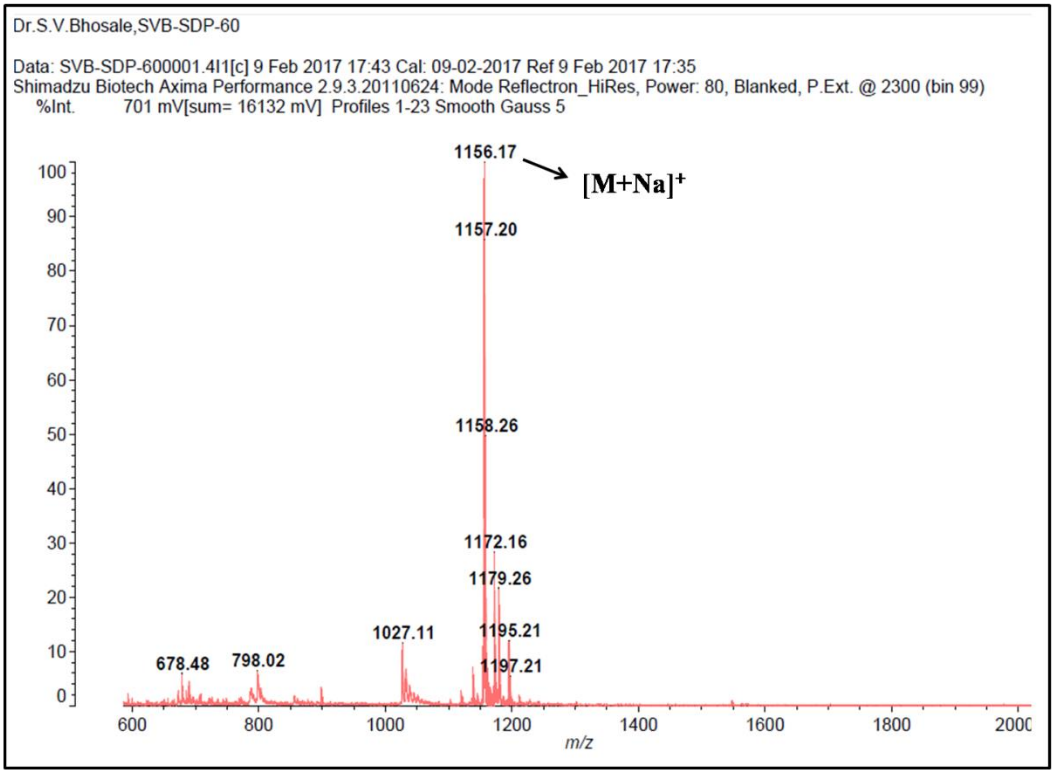

Figure S4. MALDI-TOF spectra of **NDI-CA**.

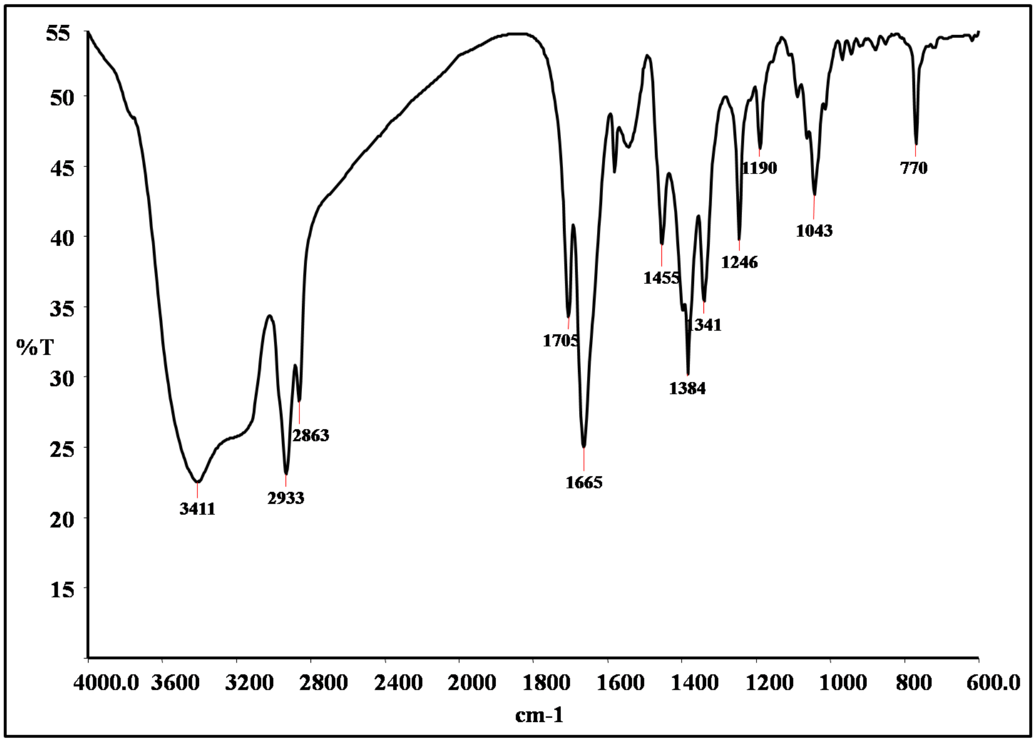

Figure S5. FT-IR spectra of **NDI-DCA**.

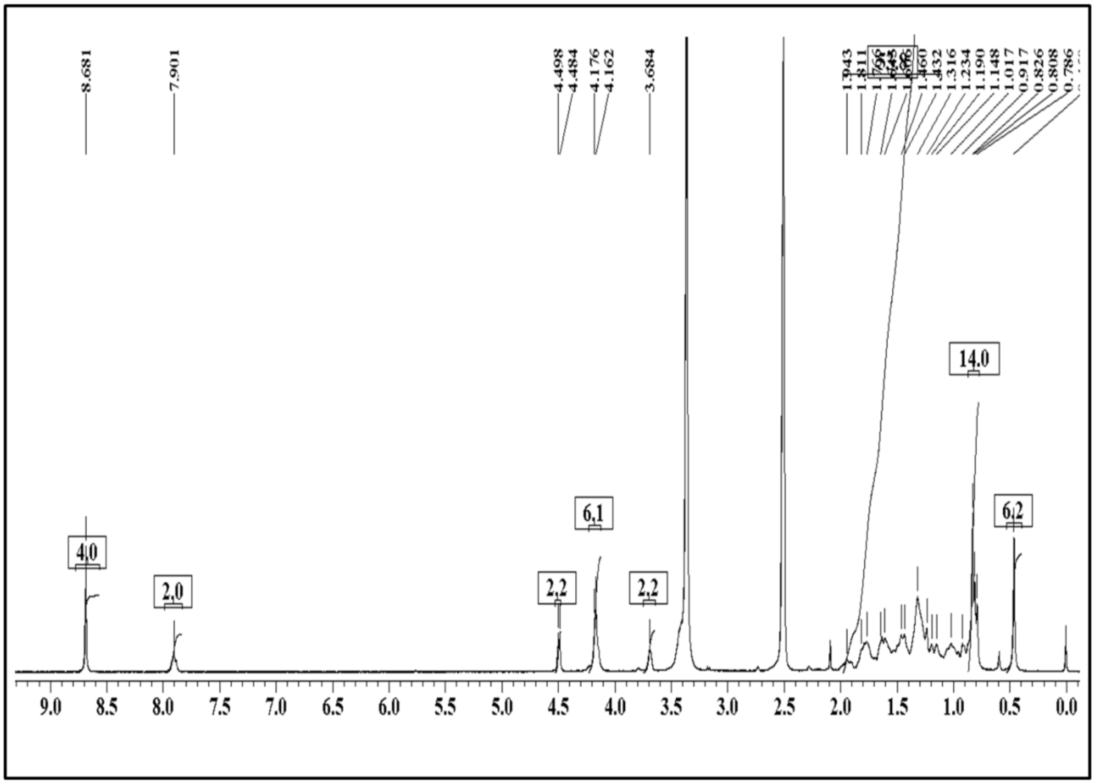

Figure S6. ^{1}H NMR spectra of **NDI-DCA**.

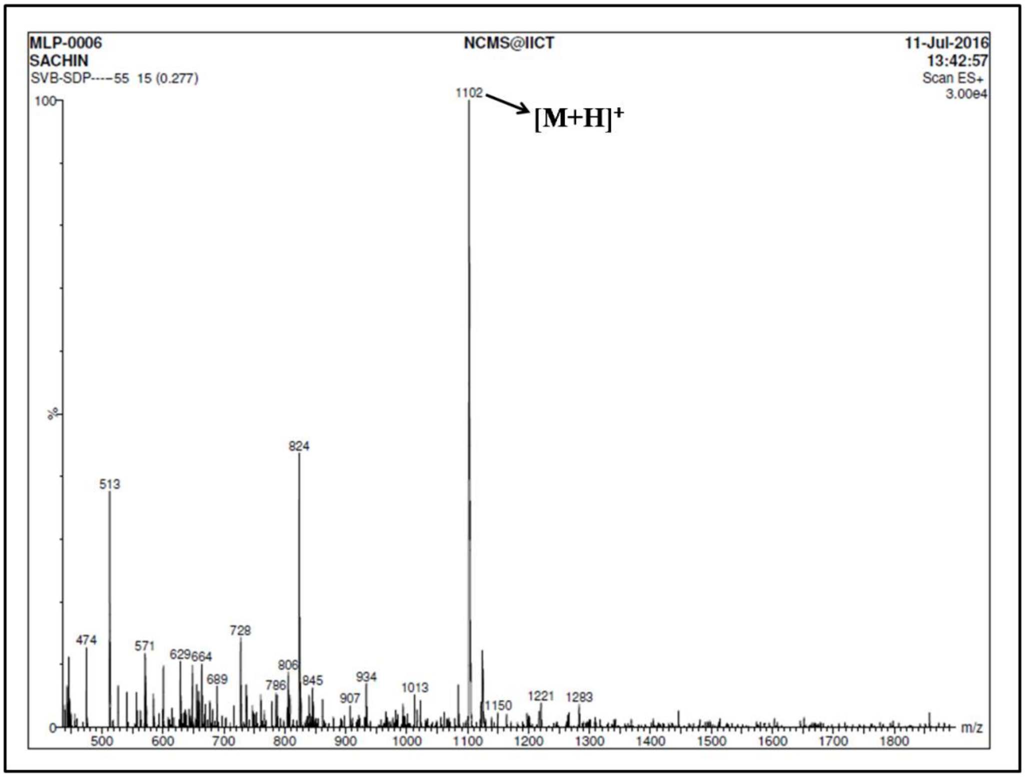

Figure S7. ESI mass spectra of **NDI-DCA**.

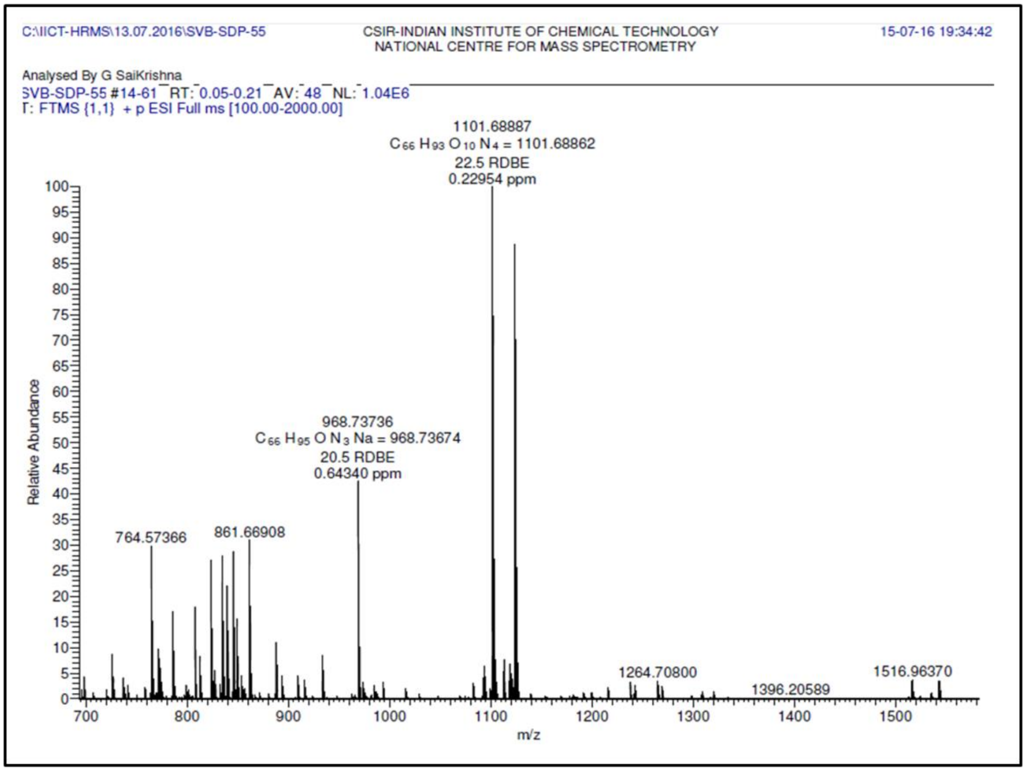

Figure S8. HRMS spectra of **NDI-DCA**.

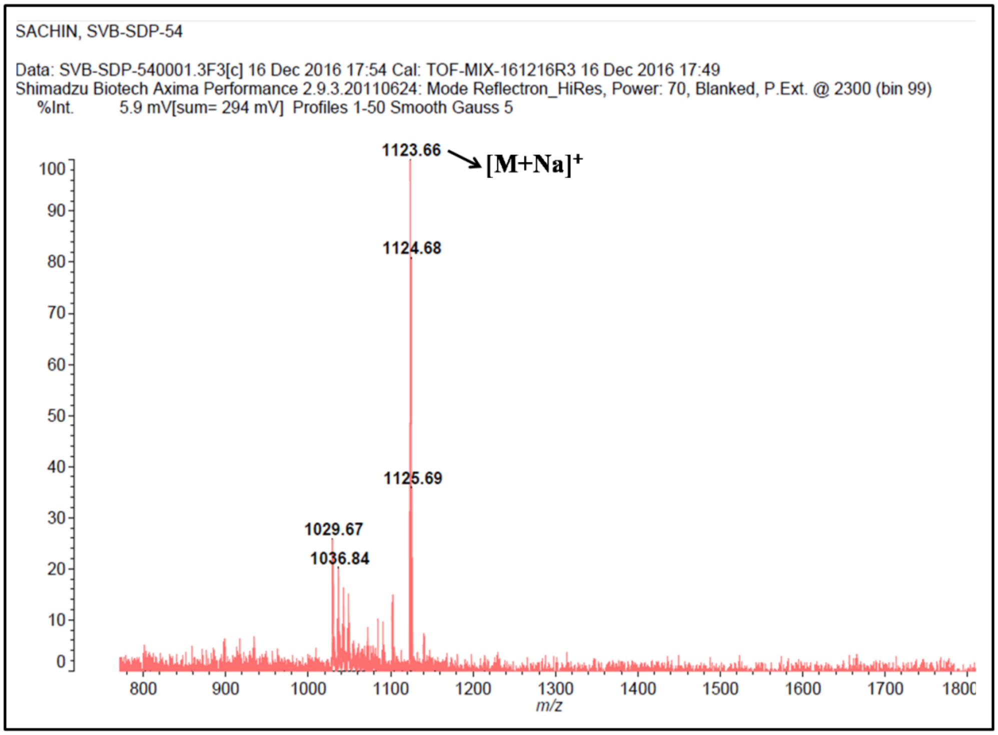

Figure S9. MALDI-TOF spectra of **NDI-DCA**.

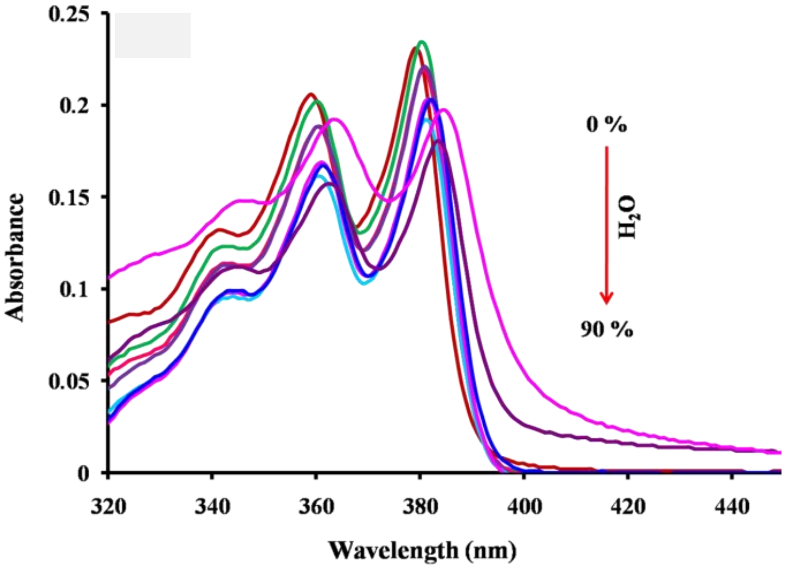

Figure 10. UV-vis spectra of NDI-CA in THF (1×10^{-5} M) upon addition of water (0-90%).

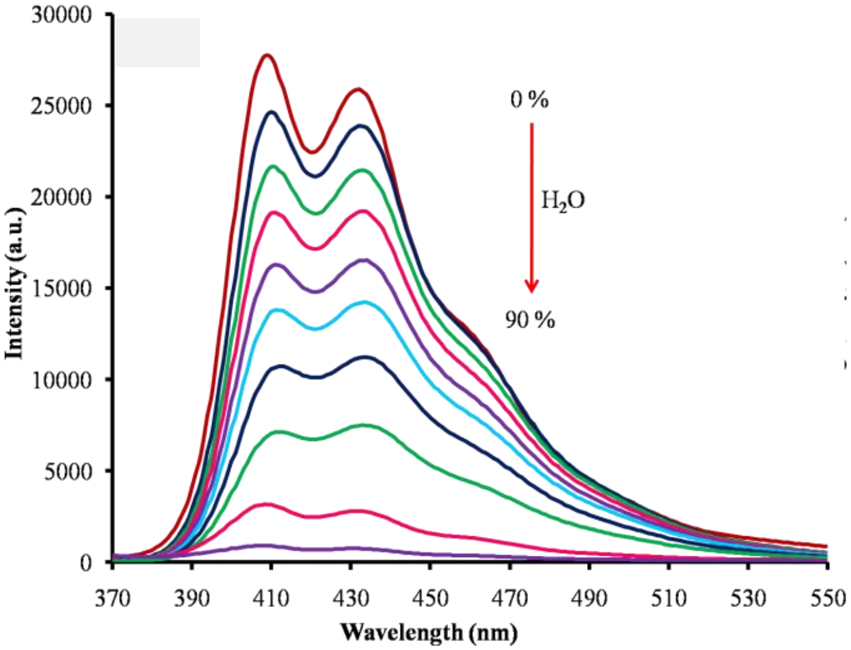

Figure S11. Fluorescence spectra of NDI-CA in THF (1×10^{-5} M) and upon addition of water (0- 90%) with λ_{ex} = 350 nm.

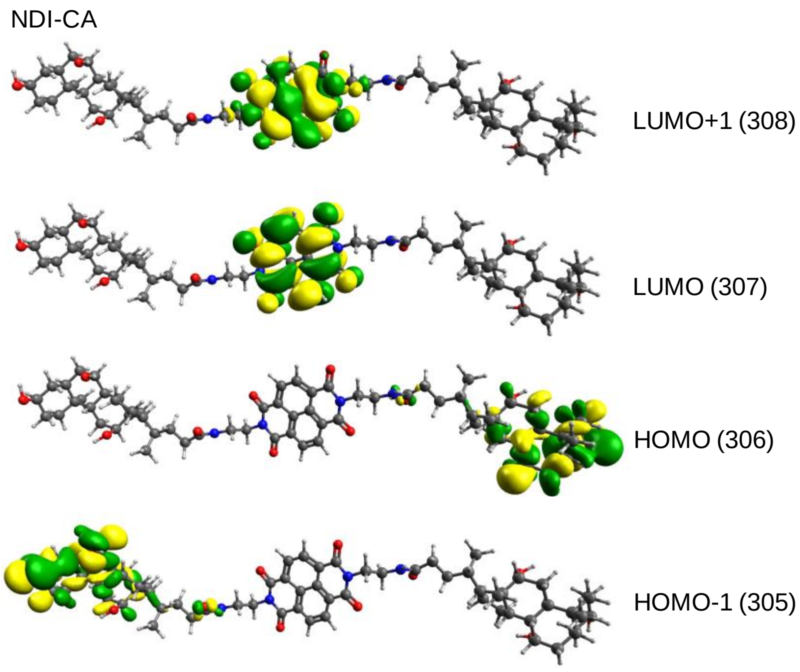

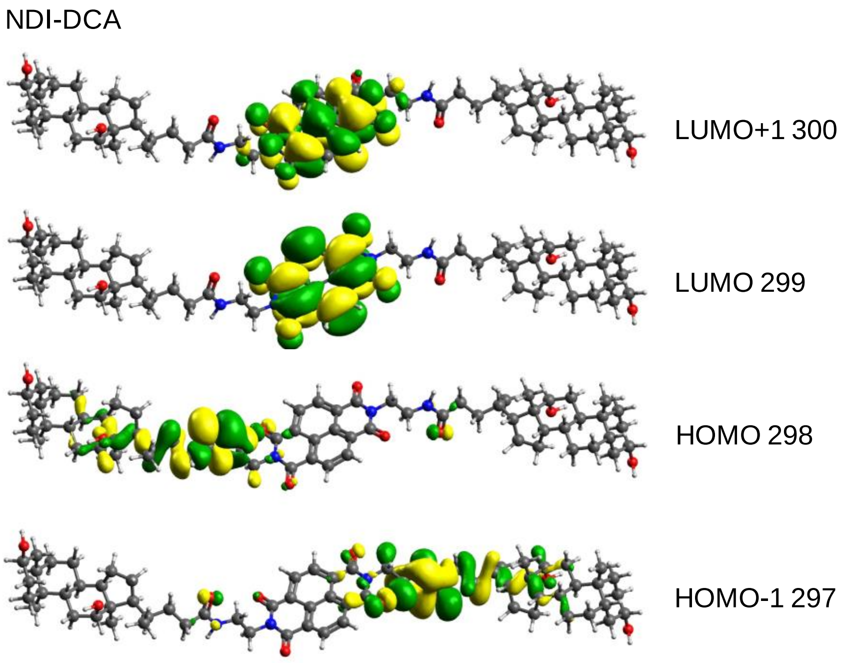

Figure S12. The frontier molecular orbitals HOMO and LUMO wave function of **NDI-CA** and **NDI-DCA** as calculated using TDDFT at B3LYP/def2-TZVP basis set.

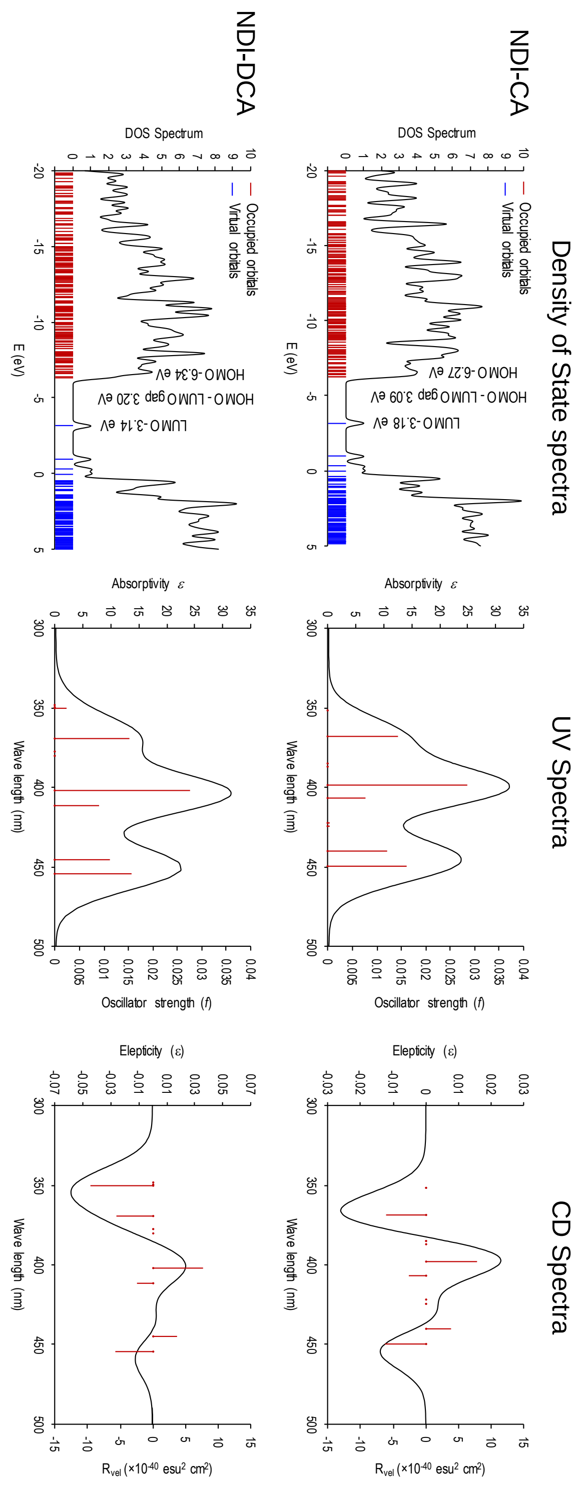

Figure S13. (A) The Density of Sate, UV and CD spectra of **NDI-CA** and **NDI-DCA** as calculated using TDDFT at B3LYP/def2-TZVP basis set. (B) A comparison between the simulated and experimental CD spectra of **NDI-DCA**.

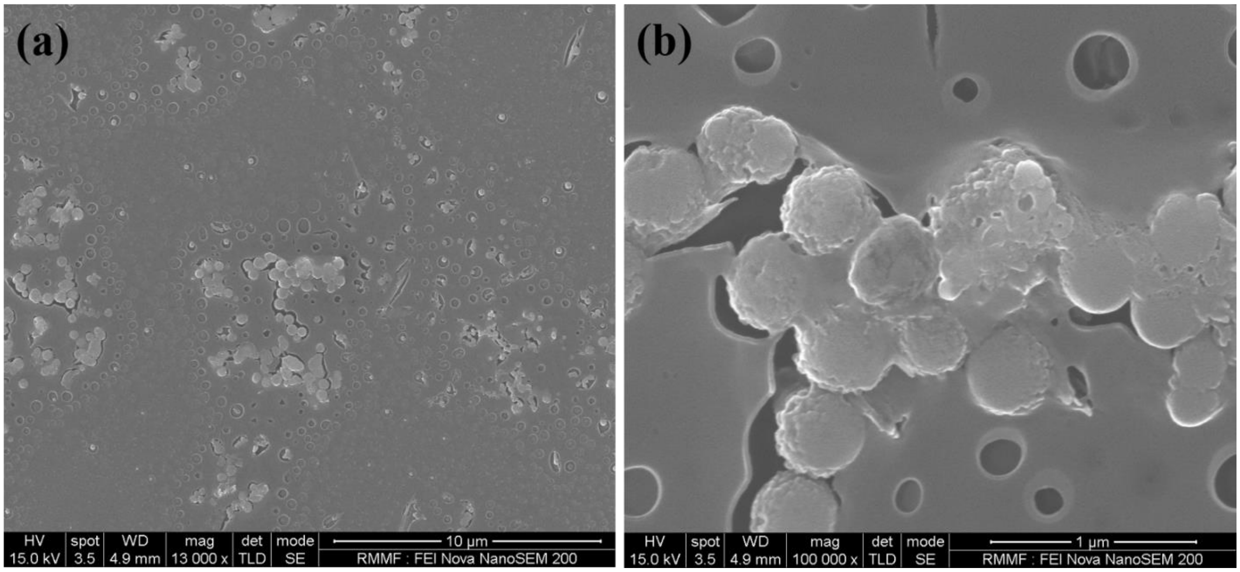

Figure S14. SEM images of **(a)** globular structures self-assembled from **NDI-CA** in THF:H_2O (20:80%); (b) zoomed image.

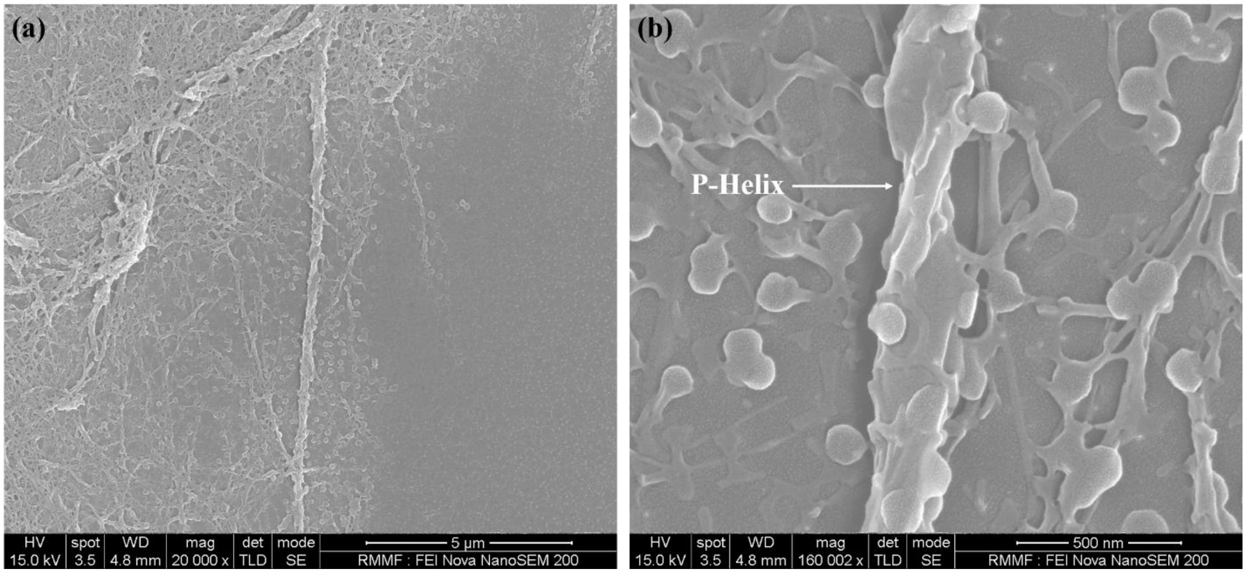

Figure S15. SEM images of **(a)** super-helical structures and spheres self-assembled from **NDI-DCA** in THF:H_2O (30:70%); (b) zoomed image.

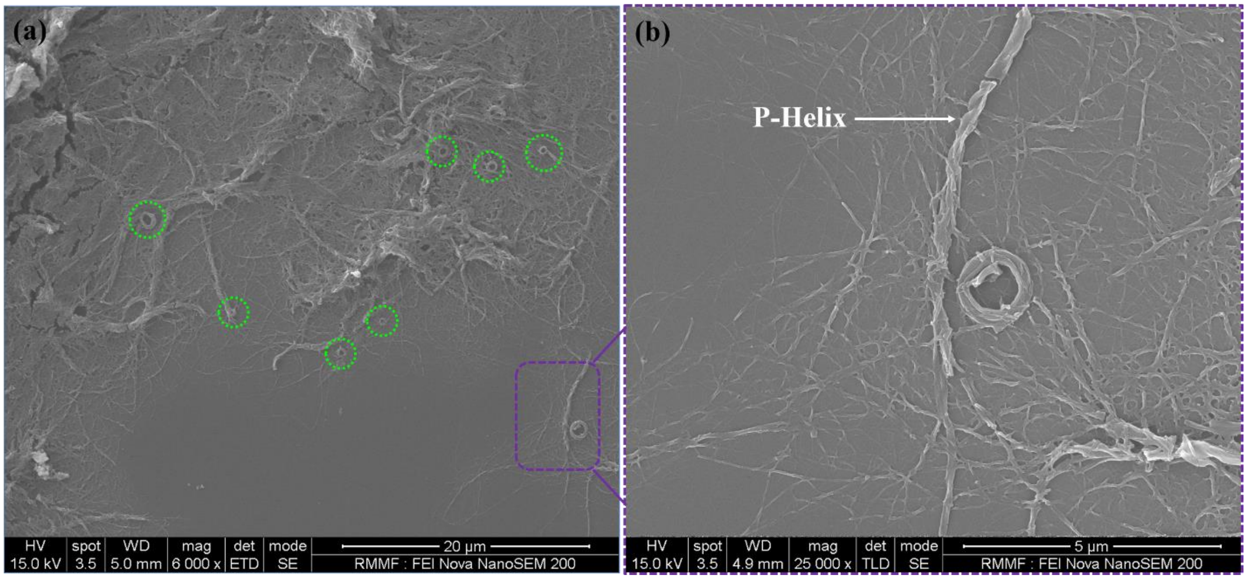

Figure S16. SEM images of **(a)** super-helical structures and spheres self-assembled from **NDI-DCA** in THF:H_2O (25:75%); (b) zoomed image.

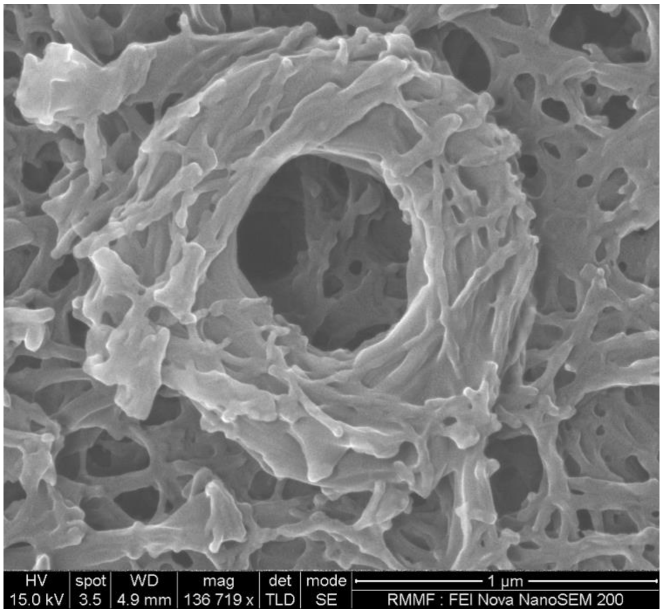

Figure S17. SEM zoomed image of ring structure self-assembled from **NDI-DCA** in THF:H_2O (25:75%).

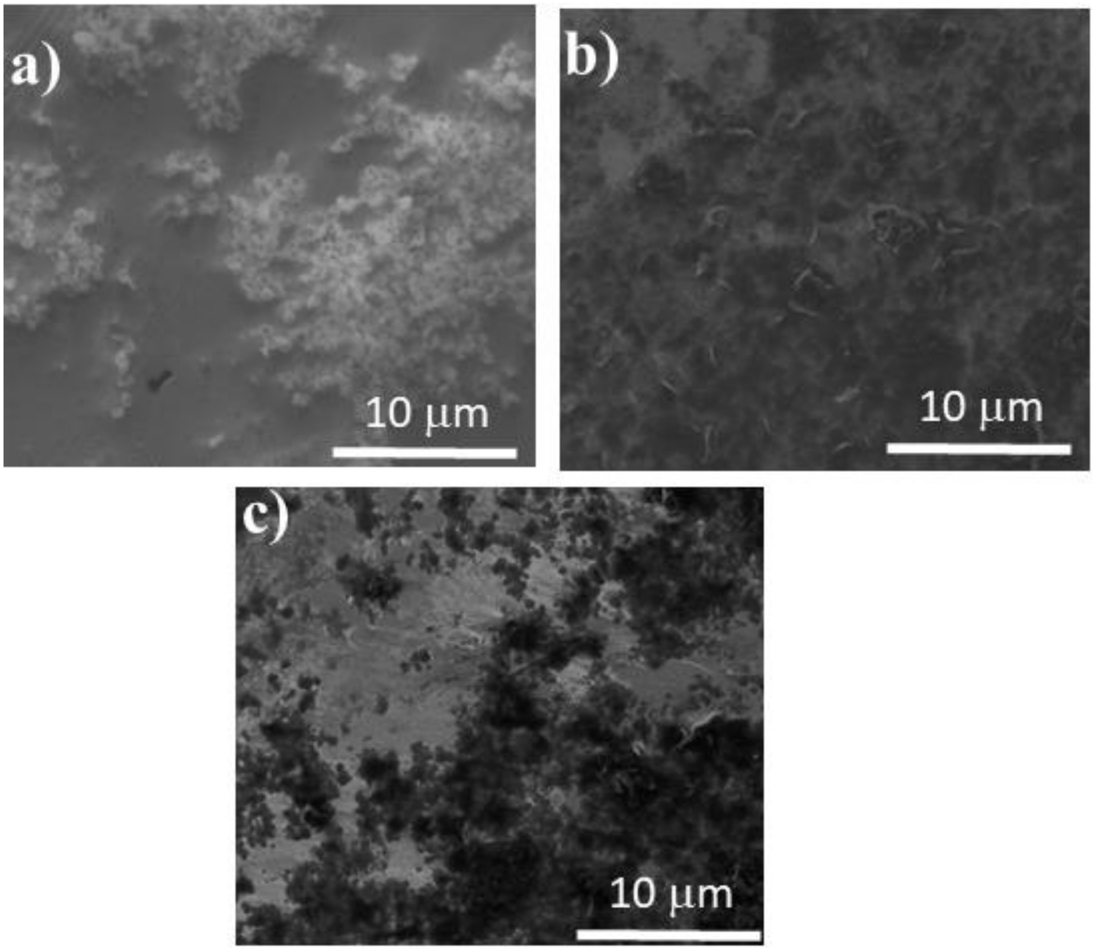

Figure S18. SEM images **NDI-DCA** (conc.10^{-4} M) in: a) THF:MeOH (20:80%), b) THF:hexane (20:80%), and c) ACN:H_2O (20:80%).

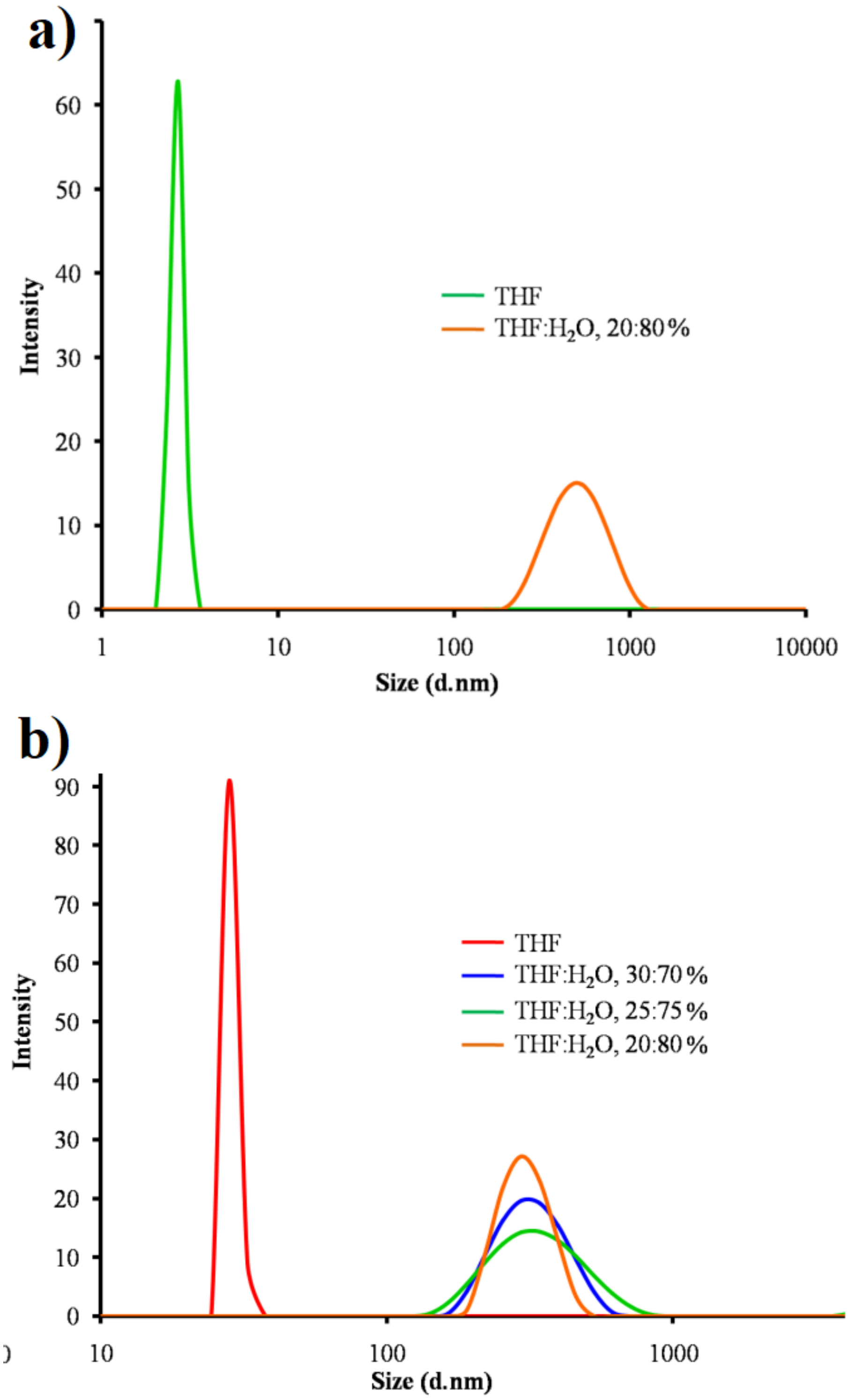

Figure S19. DLS measurements of (a) **NDI-CA** and (b) **NDI-DCA** in various ratios of THF:H_2O.

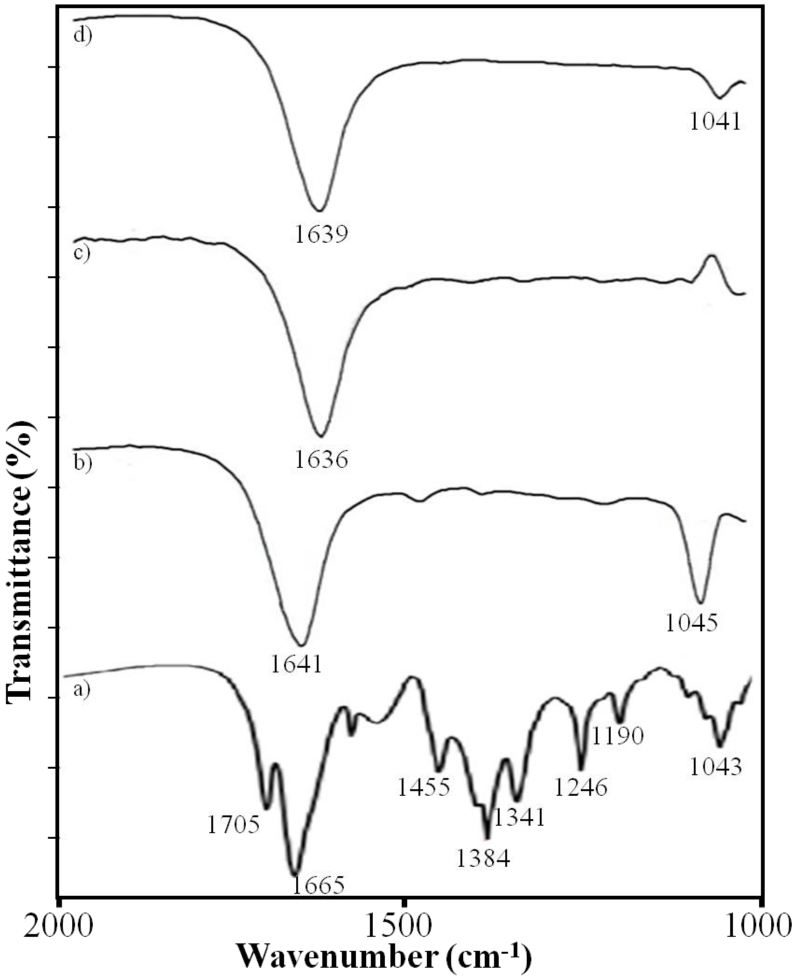

Figure S20. FT-IR spectra of NDI-DCA in: (a)THF, (b) THF: water (40:70%); (c) THF: water (40:75%) and (d) THF: water (20:80%)

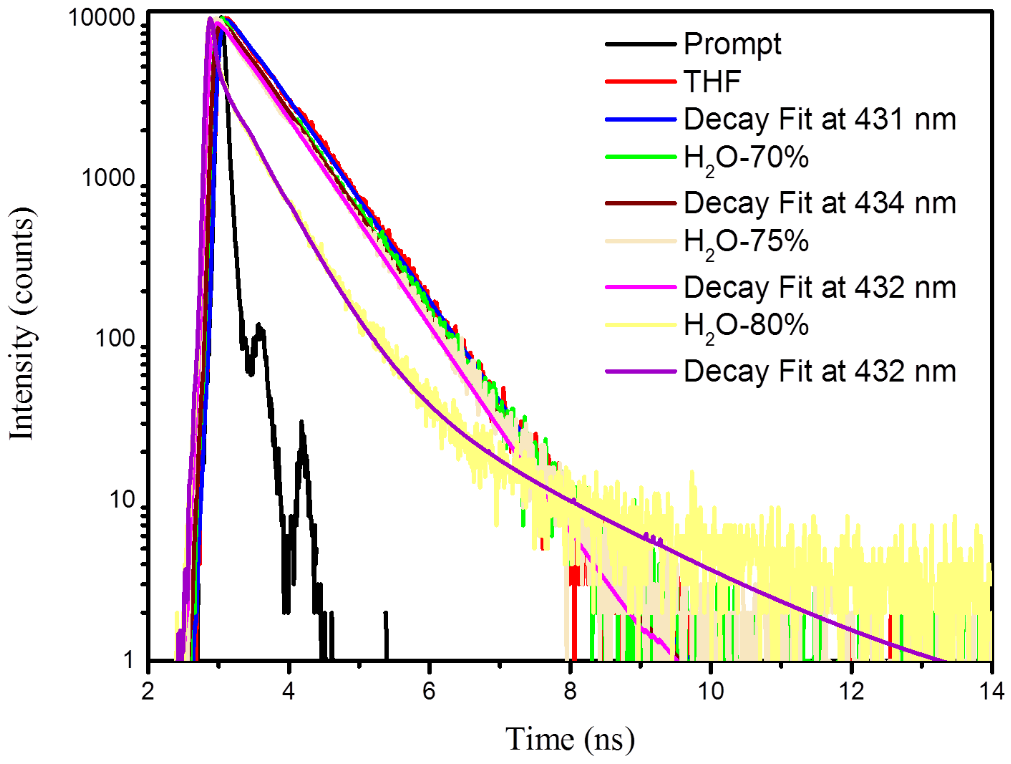

Figure 21. Fluorescence Lifetime measurement of NDI-DCA in various ratios of water in THF, respectively.

Table S1. Fluorescence life time of measurement **NDI-DCA** in the various concentrations of water in THF, respectively.

Sample Code	τ_1 (ns)	Contribution (%)	τ_2 (ns)	Contribution (%)	τ_3 (ns)	Contribution (%)
THF	0.965	100	-	-	-	-
H_2O-70%	0.949	100	-	-	-	-
H_2O-75%	0.937	100	-	-	-	-
H_2O-80%	0.712	26.16	0.004	71.36	2.67	2.48
